# *β-catenin^C429S^* mice exhibit sterility consequent to spatiotemporally sustained Wnt signalling in the internal genitalia

**DOI:** 10.1038/srep06959

**Published:** 2014-11-07

**Authors:** Takuya Murata, Yuichi Ishitsuka, Kumiko Karouji, Hideki Kaneda, Hideaki Toki, Yuji Nakai, Shigeru Makino, Ryutaro Fukumura, Hayato Kotaki, Shigeharu Wakana, Tetsuo Noda, Yoichi Gondo

**Affiliations:** 1 Mutagenesis and Genomics Team, RIKEN BioResource Center, Tsukuba, Ibaraki, Japan; 2 Population and Quantitative Genomics Team, RIKEN Genomic Sciences Center, Yokohama, Kanagawa, Japan; 3 Japan Mouse Clinic, RIKEN BioResource Center, Tsukuba, Ibaraki, Japan; 4 Team for Advanced Development and Evaluation of Human Disease Models, RIKEN BioResource Center, Tsukuba, Ibaraki, Japan

## Abstract

Wnt/β-catenin signalling regulates numerous developmental and homeostatic processes. Ctnnb1 (also known as β-catenin) is the only protein that transmits signals from various Wnt ligands to downstream genes. In this study, we report that our newly established mouse strain, which harbours a Cys429 to Ser missense mutation in the *β-catenin* gene, exhibited specific organ defects in contrast to mice with broadly functioning Wnt/β-catenin signalling. Both homozygous mutant males and females produced normal gametes but were infertile because of abnormal seminal vesicle and vaginal morphogenesis. An ins-TOPGAL transgenic reporter spatiotemporally sustained Wnt/β-catenin signalling during the corresponding organogenesis. Therefore, *β-catenin^C429S^* should provide new insights into β-catenin as a universal component of Wnt/β-catenin signal transduction.

β-catenin acts in two independent ways: as a transcriptional cofactor of the canonical Wnt/β-catenin signalling pathway and as a mediator of cell–cell interactions^1^,^2^. Wnt/β-catenin signalling is critical for cell fate determination during embryonic development and in adult stem cells^1^,^3^. Anomalous signalling often causes developmental abnormalities and diseases, including cancer^2^,^3^.

The canonical Wnt/β-catenin signalling pathway is one of the pathways activated by Wnt^1^,^2^. Various Wnt family members are thought to activate this pathway via the same molecular mechanism of signal activation^1^,^3^, and 19 Wnt ligands have been reported in mice (http://www.stanford.edu/group/nusselab/cgi-bin/wnt/mouse). Upon Wnt ligand-mediated stimulation, β-catenin escapes from its default degradation, which is regulated by N-terminal phosphorylation and subsequent ubiquitination^1^. The C-terminus acts as the main transactivation domain^4^. Therefore, both the N- and C-termini have common essential functions independent of the activating Wnt ligand. ‘Central armadillo repeats' may fine-tune this signal as an exclusive interface with various proteins^1^; however, this switching of the partners is not yet well understood^1^,^3^.

In this study, we report the development of a *β-catenin^C429S^* mouse strain, which harbours a cysteine 429 in the seventh armadillo repeat. The ins-TOPGAL transgenic reporter^5^ visualized abnormally sustained Wnt/β-catenin signalling during organogenesis of the seminal vesicle and vagina in homozygous mutant males and females, respectively. However, little is known about the involvement of Wnt/β-catenin signalling in the seminal vesicle and vagina in contrast to knowledge concerning other reproductive organs such as the uterus^6^,^7^,^8^,^9^,^10^, oviduct^6^,^7^ and ovary^11^. These signal anomalies coincided with malformation of the corresponding organs that led to infertility despite normal gamete production. Therefore, this C429S mutation revealed that β-catenin possesses an intrinsic capacity to fine-tune Wnt/β-catenin signalling *in vivo*.

## Results

### *β-catenin^C429S^* mice were infertile despite normal sperm and oocytes productions

**Figure 1:**
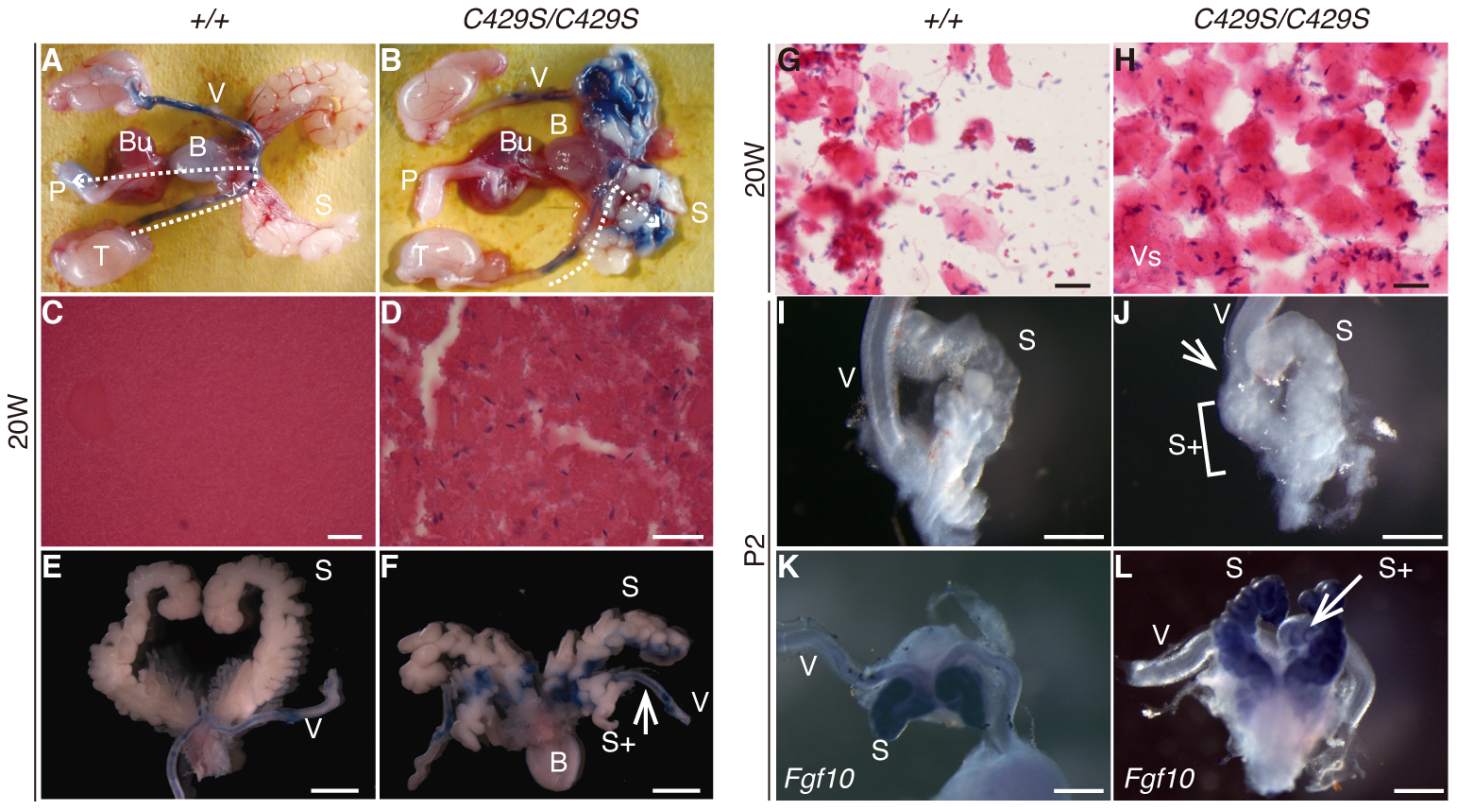
Infertility in male *β-catenin^C429S/C429S^* mice was because of abnormal seminal vesicle morphology. (A, B) Injected trypan blue visualized sperm transportation route (n = 3). The dashed white line in B indicates that the dye flowed ectopically into the seminal vesicle. (C, D) Hematoxylin & eosin (HE)-stained sections of seminal vesicles. Spermatocytes were ectopically observed only in the mutant seminal vesicles. Gross morphological views of the seminal vesicle at 20 weeks (E, F) and P2 (I, J). The very caudal end of the vas deferens is connected to the proximal side (white arrow) of the extra seminal vesicle (S+; white bracket). (G, H) Sperm were observed at near equal levels in vaginal plug smears from wild-type and homozygous females. (K, L) The duplicate seminal vesicle (S+) expresses *Fgf10*, a seminal vesicle marker gene^49^. Scale bars: 25 μm (C, D, G and H), 5 mm (E, F) and 0.5 mm (I–L). S, seminal vesicle; T, testis; V, vas deferens; P, penis; B, bladder; Bu, bulbourethral gland.

**Figure 2:**
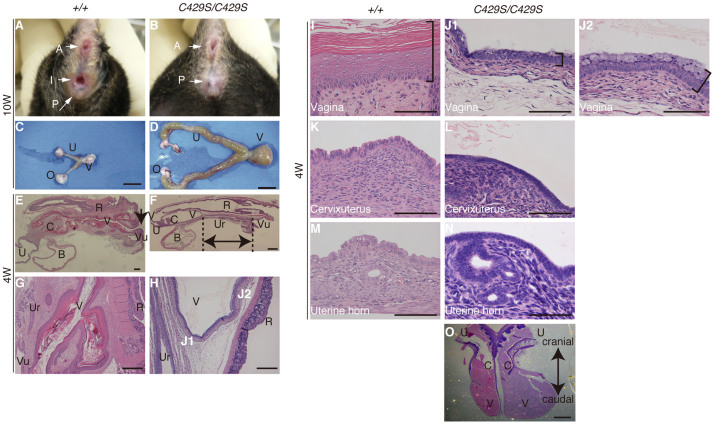
Infertility in female *β-catenin^C429S/C429S^* mice resulted from vaginal aplasia. (A, B) Missing introitus and (C, D) hydrometrocolpos at 10 weeks. (E, F) Sagittal sections at 4 weeks when vaginal canalization occurred in wild-type mice (arrow) and hydrometrocolpos did not proceed in the homozygous mice. The caudal end of the mutant vagina was distant from the vulva (double arrows). (G, H) The most caudal region of the vagina as observed at a higher magnification. Although the wild-type vagina reaches the vulva, the homozygous vagina is aplastic. (I, J1, J2) The homozygous vaginal epithelium (brackets) is thinner. Unlike the wild-type epithelium, undifferentiated columnar cells^15^ but no differentiated keratinized cells^15^ were observed in the homozygotes. The homozygous vaginal epithelial cells were heterogenous; mucous cells were observed in J2 rather than J1. The J1 and J2 region is illustrated in H. (K–N) Higher magnification images of the cervicouterine region (K, L) and uterine horn (M, N). (O) Some of the mutants exhibited separated vaginas. Scale bars: 1 cm (C, D), 1 mm (E, F), 400 μm (G), 100 μm (H), 10 μm (I–N) and 2 mm (O). A, anus; I, vaginal introitus; P, penis; V, vagina; U, uterus; C, cervix; O, ovary; B, bladder; R, rectum; Ur, urethra; Vu, vulva.

We established a novel *β-catenin^C429S^* congenic mouse strain (Fig. S1 and Tables S1, S2). Both the homozygous males and females were infertile by natural mating. However, the sperms and oocytes of the homozygotes gave rise to normal progeny by *in vitro* fertilization and embryonic transfer (Table S3). The homozygous testis and ovary were also histologically normal (Fig. S2). Only the seminal vesicle (Figs. 1A–F and Table S4) and vagina (Fig. 2 and Table S4) exhibited abnormal morphologies in a recessive and semi-dominant manner, respectively. All data hereafter are presented as comparisons between *β-catenin^+/+^* and *β-catenin^C429S/C429S^* mice. The numbers of *β-catenin^C429S^* homozygous newborns were somewhat reduced (Table S5). However, the surviving homozygotes lived for up to 2 years (male: n = 9/9, 100%; female: n = 8/8, 100%) without any obvious abnormalities except for infertility by natural mating.

### Infertility in the *β-catenin^C429S^* mice was consequent to abnormal internal genital morphologies

Male infertility (n = 16/16, 100%) was caused by drastic changes in the sperm ejection route (Fig. 1B). The sperm were ejaculated through a detour in an abnormally duplicated seminal vesicle (n = 3/3, 100%; Figs. 1D, H), and therefore may have lost their fertility. Female infertility resulted from the absence of the vaginal introitus (Fig. 2B) and subsequent hydrometrocolpos (Fig. 2D) that physically prevented natural mating (n = 10/11, 91%). Only one exceptional homozygous female (n = 1/11, 9.1%) exhibited a normal vaginal introitus and could produce pups; the remaining homozygotes exhibited vaginal aplasia. The single exceptional female suggests that fertility could be restored in homozygote females following vaginal opening. The missing introitus resulted from an aplastic vagina (Figs. 2F, H) with poorly differentiated epithelium (Figs. 2J1, 2J2) in contrast to the thick and keratinized epithelium in wild-type mice (Fig. 2I). The homozygous cervix and uterus were normal (Figs. 2L, N).

### Anomalous Wnt/β-catenin signalling was observed only in seminal vesicle organogenesis in males

**Figure 3:**
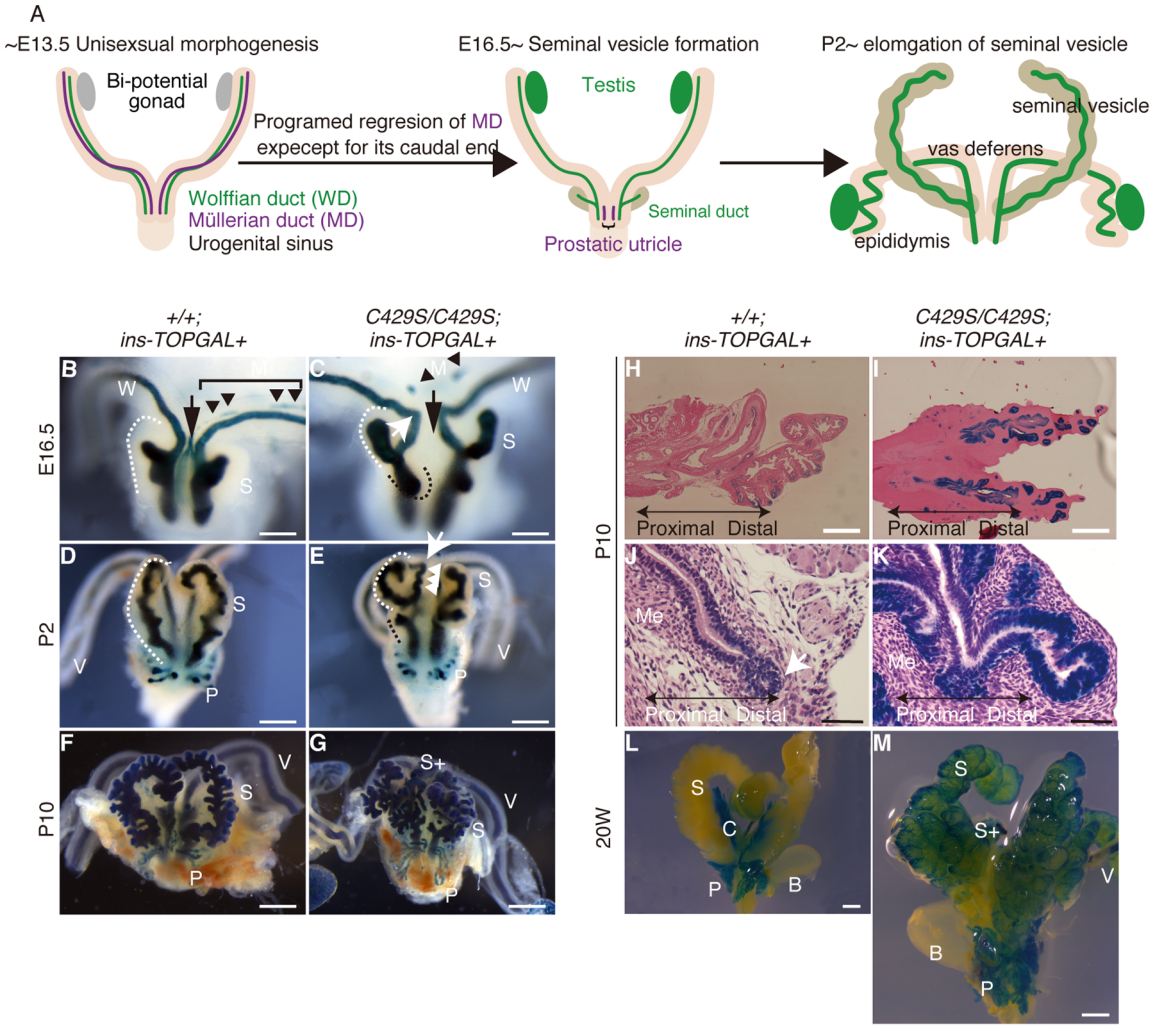
Wnt/β-catenin signalling is sustained during seminal vesicle organogenesis. (A) A schematic illustration of normal seminal vesicle formation^13^. A male-specific hormone promotes Müllerian duct regression. Subsequently, the seminal vesicle buds from the caudal Wolffian ducts. (B, C) At E16.5, during seminal duct budding, the Wolffian duct became abnormally bent at a right angle (white arrow). Extra buds were also observed (black bracket). (D–G) Ins-TOPGAL visualized abnormal seminal vesicle formation. (D, E) These abnormalities were observed at the later P2 stage. The bent Wolffian duct exhibited a wavy shape, similar to a normal seminal vesicle. (F, G) X-gal staining revealed abnormal seminal duct branching at P10. This stained portion corresponded to the *Fgf10*-positive (Fig. 1L) duplicate seminal vesicle (Fig. 1J). (H, I) The ins-TOPGAL expression was uniform and distally localized in the homozygous and wild-type seminal ducts, respectively, at P10 in eosin counterstained section. (J, K) The homozygous mesenchymal cells were vastly increased adjacent to the seminal duct at P10. (L, M) In adult mice (20 week), ins-TOPGAL was expressed in the homozygotes but not in the wild-type mice. Scale bars: 0.2 mm (B, C, H and I), 0.5 mm (D, E) and 2 mm (F, G, J–M). S, seminal vesicle; S+, duplicated seminal vesicle; V, vas deferens; P, prostate; C, coagulating gland; W, Wolffian duct; Me, mesenchymal cells; M, regressing Müllerian duct.

**Figure 4:**
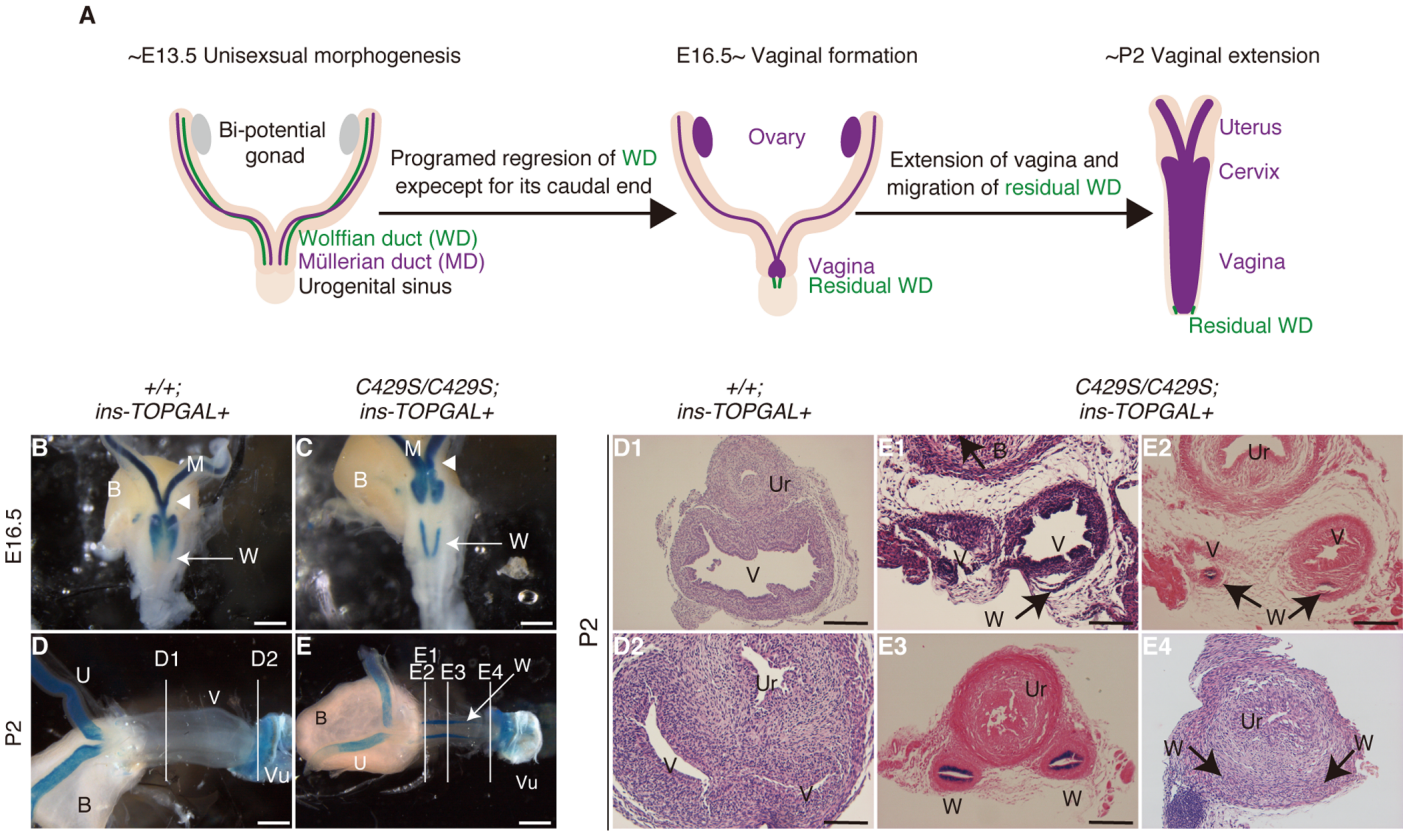
Wnt/β-catenin signaling is sustained during vaginal organogenesis. (A) Schematic illustration of normal vaginal formation. In the absence of male hormones, all but the caudal ends of the Wolffian ducts regress^22^. The caudal Müllerian ducts fuse and extend as the vagina^15^. The residual Wolffian duct caudally leads the vagina^15^,^22^. (B, C) At E16.5, X-gal staining revealed unfused Müllerian ducts (white arrowhead). The vagina became ins-TOPGAL negative in both the wild-type and homozygous mice at a later stage. (D, E) At P2, although the wild-type vagina reaches the vulva, the unfused homozygous vagina does not extend. Ins-TOPGAL revealed abnormally elongated Wolffian ducts in the homozygotes (white arrow). White lines indicate the planes of the transverse sections (D1, D2 and E1–E4). The caudal direction is to the right. (D2) In wild-type mice, no obvious ins-TOPGAL positive staining was observed at the most distal end of the vagina where the residual Wolffian duct should exist^15^. (E1–E4) Using the ins-TOPGAL signal, the Wolffian duct epithelium was dorsolaterally observed within the stromal wall of the septate vagina (E1, E2) at the cranial side. At the caudal side, only the Wolffian duct was observed (E3, E4). Near the vulva, the Wolffian duct was rather faint (E4). Scale bars: 2 mm (B–E), 0.2 mm (D1) and 0.1 mm (D2, E1–E4). V, vagina; U, uterus; Vu, vulva; B, bladder; Ur, urethra; W, residual Wolffian duct.

We used ins-TOPGAL reporter transgenic mice^5^ to visualize Wnt/β-catenin signalling *in vivo* during organogenesis of the seminal vesicle and vagina. As shown in the previous report^12^, we observed normal Wnt/β-catenin signalling activity in the wild-type seminal vesicle and vagina (Figs. 3, 4). During normal organogenesis, different reproductive ducts, i.e., the Wolffian^13^,^14^ and Müllerian ducts^15^,^16^, develop into various internal genital organs along the cranial–caudal axis in males and females, respectively. The seminal vesicle (Fig. 3A) and vagina (Fig. 4A) originate from the very caudal portions of the respective ducts^13^,^15^.

During initial seminal vesicle formation in embryonic day 16.5 (E16.5) homozygotes, ins-TOPGAL revealed not only the normal seminal vesicle bud (white bracket) but also an extra bud (black bracket) and an abnormal bend at the very caudal end of the Wolffian duct (white arrow; n = 2/2, 100%; Fig. 3C). The morphological analysis (n = 2/2, 100%; Fig. 1J) and *in situ* hybridization with a seminal vesicle marker (n = 3/3, 100%; Fig. 1L) revealed that the caudal end-bent Wolffian duct (Figs. 3C, E) developed into the duplicated seminal vesicle (Fig. 1F). Because the caudal Wolffian duct is known to differentiate into the caudal vas deferens under normal conditions^13^, a cell fate change occurred in the male homozygotes alone at this limited time point and place. Later in development (P10), ins-TOPGAL revealed an excessively branched seminal duct (n = 3/3, 100%; Fig. 3G). Ins-TOPGAL was uniformly expressed along the proximal–distal axis of the homozygous seminal duct (n = 3/3, 100%; Fig 3I) in contrast to the distal localization observed in the wild-type duct (Fig. 3H), and the adjacent mesenchymal cells in the former had abnormally proliferated (n = 3/3, 100%; Fig. 3K). *β-catenin* regulates branch morphogenesis^17^, e.g., *β-catenin* conditional knockout mice were found to exhibit reduced branching in the lung^18^ and kidney^19^,^20^. In peculiar, the *β-catenin^C429S/C429S^* abnormalities were limited to seminal vesicle, whereas other organs such as the lung and kidney (n = 4/4, 100%; Fig. S3) remained normal. At 20 weeks, sustained ins-TOPGAL expression was observed in both the original and duplicated seminal vesicles in the homozygotes (n = 3/3, 100%; S+ in Fig. 3M), whereas this expression had already terminated in the wild-type seminal vesicle (Fig. 3L).

### Sustained Wnt/β-catenin signalling was observed in the residual reproductive duct near the vagina

In the homozygous females, abnormally sustained ins-TOPGAL expression was also observed near the vagina rather than in the vagina itself. Ins-TOLGAL was abnormally expressed where the residual Wolffian duct verged caudally on the vagina (Figs. 4B–E). The mutant Wolffian ducts first regressed normally (n = 2/2, 100%; Fig. 4C), after which the residual Wolffian duct elongated abnormally (n = 4/4, 100%; Fig. 4E). Arguments regarding the origin of the vagina with respect to the role of the Müllerian duct have recently settled down following the use of lineage tracing in mice^21^ (Fig. 4A); however, the role of the residual Wolffian duct in vaginal formation remains controversial^15^. A previous lineage tracing study clearly showed that the vagina derived solely from the caudal Müllerian duct and included neither the caudal residual Wolffian duct nor urogenital sinus, both of which were near the caudal end of Müllerian duct^21^. Three-dimensional histological analyses conducted during several stages of vaginal formation suggested that the caudal residual Wolffian duct played an assistive role^22^. In our study, the poorly elongated vagina and abnormally elongated Wolffian duct observed in the *β-catenin^C429S/C429S^* mice suggested role(s) for the wild-type Wolffian duct in vaginal formation. For instance, during normal development, the Wolffian duct may act as a guide for vaginal elongation. In the homozygotes, the abnormally elongated Wolffian duct may have indirectly prevented vaginal elongation, leading to vaginal aplasia.

Although the morphological defects were hyperplastic in the seminal vesicle and hypoplastic in the vagina, the caudal Wolffian ducts were commonly hyperplastic in both sexes. As morphologically distinctive differences in the Wolffian ducts appeared, specifically excessive branching in males and simple elongation in females, the hyperplastic defects in the Wolffian ducts may occur after sex determination.

### The transcription cofactor activities of the wild-type and C429S β-catenin proteins were almost equivalent

To determine whether the wild-type and C429S β-catenin proteins differed in terms of transcription cofactor activity^1^, we conducted a assay in which we cotransfected the wild-type or C429S *β-catenin* cDNA with the canonical Wnt/β-catenin TOP-FLASH reporter plasmid^23^ into HEK293 cells, which have been popularly used in TOPFLASH assays^24^,^25^. The luciferase assay results (n = 3) indicated that the cofactor activities of the β-catenin^C429S^ and wild-type β-catenin proteins were equivalent (Fig. S4).

In summary, the infertility observed in *β-catenin^C429S/C429S^* mice resulted from abnormal organogenesis of the seminal vesicle and vagina. In the homozygous mutants, sustained Wnt/β-catenin signalling was spatiotemporally limited to the very caudal Wolffian ducts of both sexes during organogenesis (Fig. S5).

## Discussion

Mice bearing a previously reported null mutation of the *β-catenin* gene exhibited developmental deficits at a much earlier gastrulation stage during embryogenesis^26^. Through the use of diverse tissue-specific Cre-drivers, *β-catenin* was also found to be essential in the organogenesis of various organs (Table 1 by Grigoryan et al^27^), including the urogenital and external genital organs^28^,^29^. Unlike the previously reported conditional knockout mice, *β-catenin^C429S^* mice, which lack Cre drivers and express β-catenin via the endogenous promoter, exhibited a unique pattern of infertility resulting from specific defects in the organogenesis of the seminal vesicle (Fig. 1) and vagina (Fig. 2), although the sperm and oocytes were normal (Table S3). While sperm *per se* does not seem to require the *β-catenin* function, the mouse strains with constitutive Wnt/β-catenin signaling in Sertoli cells had defects with the disrupted spermatogenesis and the retained uterus^30^,^31^. The Wnt4/β-catenin pathway is indispensible for female sex determination^32^,^33^ and is also essential for subsequent ovary differentiation^11^. Regarding reproductive ducts, activation of the Wnt/β-catenin signalling in Wolffian ducts using MMTV-Cre did not affect the organogenesis such as seminal vesicle, vas deferens, and epididymis^34^. Although the uterus has been the most implicated organ associated with Wnt/β-catenin signalling in the Müllerian ducts or their derivatives^6^,^7^,^8^,^9^,^10^,^35^, little is known about the involvement of Wnt/β-catenin signalling in the organogenesis of vagina. Our novel *β-catenin^C429S^* mutant strain is the only mouse strain with deficits in both the seminal vesicle and vagina, suggesting the potential involvement of Wnt/β-catenin signalling. This mutant strain will serve as a good resource to elucidate these organogenic processes and Wnt/β-catenin signal transduction.

The structural prediction software programs Sift^36^ and PolyPhen2^37^ predicted the C429S substitution in β-catenin as ‘affecting protein function' and ‘possibly damaging', respectively. Cocrystal structural studies also indicated that cysteine 429 was located on the surface of a groove constructed from 12 armadillo repeats^38^,^39^,^40^. This groove was shown to act as a common interface with various proteins, including APC and Tcf, which are essential components of the broad Wnt/β-catenin signal function/expression^1^. In contrast to the broad expression of APC, Tcf, and the Wnt/β-catenin signalling pathway, the phenotypes in the *β-catenin^C429S/C429S^* mice were highly restricted to particular organs, such as the seminal vesicle and vagina. Therefore, the phenotypes associated with the *β-catenin^C429S/C429S^* genotype cannot be explained in terms of APC or Tcf. If β-catenin^C429S^ binding to APC and Tcf was the key deficit resulting from the C429S substitution, the phenotypes in the *β-catenin^C429S/C429S^* mice should be broader and not limited to the seminal vesicle and vagina. Therefore, to exhibit this limited phenotype, the interactions of the β-catenin^C429S^ protein with APC and Tcf must be retained. The transcription cofactor activities, in which APC and Tcf are essential^1^,^2^, were nearly equivalent between the wild-type and C429S β-catenin proteins (Fig. S4). Rather, the conformation change induced by C429S must affect an aspect of the β-catenin protein other than its essential interactions with APC and Tcf. Recent studies have also shown that the phosphorylation of some Ser/Thr and Tyr residues in the armadillo repeats may dynamically regulate the function of β-catenin^1^. Therefore, the *β-catenin^C429S^* mouse may be a good resource for analysing the molecular mechanisms of the central armadillo repeats to identify and elucidate the unknown interacting counterpart(s) that may contribute to the fine-tuning of Wnt/β-catenin signalling.

As the human and mouse β-catenin proteins share 100% amino acid sequence identity, *β-catenin^C429S^* may serve as a new model of human infertility. Many studies have been reported particularly focusing on the N-terminus, which is a well known hot spot for tumorigenesis^41^. Regarding the importance of the cysteine 429 residue *in vivo*, the C429G (Melanoma; TCGA-D3-A1Q5) and C429Y (not recorded; COSM1235320) mutations have also been recorded in the cBioportal (http://www.cbioportal.org/public-portal/index.do) and COSMIC (http://cancer.sanger.ac.uk/cancergenome/projects/cosmic/) human cancer databases, respectively. On the other hand, human *β-catenin* has hardly been anticipated as a risk factor for infertility; only one research group has attempted such a study but could not find any such mutations^42^ in patients with the Mayer–Rokitansky–Küster–Hauser syndrome (OMIM 277000). However, in that survey^42^, the authors focused only on the hot spot N-terminus for tumorigenesis^41^. Some *WNT* ligand genes have been found to be crucial for infertility, such as *WNT4* (OMIM 603490) in the Serkal syndrome (OMIM 611812). Regarding the phenotypes in mice, vaginal aplasia (Fig. 2B) and separated caudal Müllerian ducts (Fig. 2I) similar to those observed in the *β-catenin^C429S^* females did exist in human diseases^43^, such as the Antley–Bixler (OMIM 207410), Bardet–Biedl (OMIM 209900), Fraser (OMIM 219000) and Winder syndromes (OMIM 267400). Therefore, it may be worthwhile to survey the *β-catenin* genotypes in these patients. We strongly believe that the C429S and other mutations in the *β-catenin* gene should be examined in human cohort studies.

In conclusion, the C429S mutation has revealed new biological functions of Wnt/β-catenin signalling. The *β-catenin^C429S^* and 11 other mouse strains are currently available from the RIKEN BioResource Center (Table S2).

## Methods

### *β-catenin* gene mutation screening

A high-throughput mutation screening of ENU-mutagenized male genomic DNA library was performed as described previously^44^,^45^,^46^. Four pairs of PCR primers were used for the screening and facilitated the multiplex amplification of exons 3, 5, 8–9 and 11 of the *β-catenin* gene (Fig. S1A and Table S1). Twelve point mutations were identified from approximately 7,400 independent G1 genomic DNA samples; missense (nonsynonymous), 6; nonsense, 1; synonymous, 2 and intronic, 3 (Figs. S1B, S1C and Table S2).

### Mouse recovery from stored frozen sperm

The mouse line harbouring the C429S amino acid substitution mutation was recovered from frozen sperm using conventional *in vitro* fertilization and embryo transfer methods. The congenic C429S *β-catenin* strain was then established on the C57BL/6J background by backcrossing over 20 generations. No other mutations were detected in any of the coding exons with the flanking *β-catenin* gene introns, as determined using the primers listed in Table S1.

### Mice

C57BL/6J mice were purchased from CLEA Japan (Tokyo, Japan). Prof. Yumiko Saga at the National Institute of Genetics, Japan kindly provided the ins-TOPGAL (RBRC02228) mouse strain^5^. Congenic ins-TOPGAL mice were generated on the C57BL/6J background by backcrossing over 15 generations. This congenic strain was deposited in the RIKEN BioResource Center (RBRC05918). All mouse experiments were conducted in accordance with the Regulations for the Animal Experiments of RIKEN. Our experimental protocols were approved by the Animal Experiments Committee of the RIKEN Tsukuba Institute.

### Male infertility testing

The *β-catenin^C429S/C429S^* males (n = 16) were mated with C57BL6/J females for >1 month.

### Dye tracing test

The sperm transportation route was traced using the trypan blue dye. The dye was injected using a 27-gauge needle into the vas deferens of sacrificed 20-week-old mice following lower abdominal incision and exposure. The genital tracts were then removed by cutting the vas deferens, urethra and ureters. The seminal vesicles were further treated in the Hank's CMF solution containing 1% collagenase to remove the surrounding membranes^47^.

### Vaginal plug smears

Vaginal plugs were collected with forceps. Each plug was suspended in 1× phosphate-buffered saline (PBS) and subsequently smeared on a glass slide. The samples were allowed to dry naturally and were stained with hematoxylin and eosin.

### Genotyping

*β-catenin^C429S^* was genotyped using the TaqMan® probe system (Life Technologies, Carlsbad, CA, USA). The following primers and probes were designed on the manufacturer's web site (https://www5.appliedbiosystems.com/tools/cadt/): F primer, 5′-CCTGTGCAGCTGGAATTCTCT-3′ and R primer, 5′-CTTGGCACACCATCATCTTGTTTT-3′; WT probe (VIC), 5′-CTAACCTCACTTGCAATAA-3′ and MT probe (FAM), 5′-CTAACCTCACTAGCAATAA-3′. PCR was performed under the conditions suggested by the manufacturer.

### *In situ* hybridization

*In situ* hybridization was performed according to standard protocols. The lower reproductive tracts (LRT) of foetal males were dissected and fixed overnight in PBS containing 4% PFA. LRTs were the dehydrated and stored in 100% methanol at −20°C until genotyping was completed. Prof. Nobuyuki Itoh of Kyoto University kindly provided the *Fgf10* probe.

### X-gal staining

LRTs were fixed for 10 min in a fixative buffer (4% formalin, 0.5% glutaraldehyde and 0.1% NP40 in PBS). After rinsing twice with PBS, LRTs were incubated overnight in X-gal buffer (4 mM K_3_Fe(CN)_3_, 4 mM K_4_Fe(CN)_6_, 1 mM MgCl_2_ and 1 mg/ml X-gal in PBS), at 37°C on a rocking platform. The samples were subsequently fixed again in 15% neutral buffered formalin. Some specimens were cleared in SCALEVIEW-A2 regent (Olympus Corporation, Tokyo, Japan) for 1 week. Other specimens were rinsed with PBS and used for paraffin sectioning.

### Microscopy

Whole-mount LRT specimens were photographed on a Leica M165FC microscope equipped with a DFC310FC digital camera and Application Suite software (ver. 3.3.0; Leica Camera AG, Solms, Germany). Histological sections were photographed on an Olympus AX80 microscope equipped with a DP50 digital camera and Studio Lite software (ver. 1.0; Olympus corporation). Some images were merged and trimmed using Photoshop CS6 (Adobe Systems Incorporated, San Jose, CA, USA).

### Structural prediction software

We used the structural prediction software programs Sift^36^ and PolyPhen2^37^, both of which can predict structural or functional damage caused by amino acid substitutions. Sift (http://sift.jcvi.org/www/SIFT_enst_submit.html) assigned a score of 0.03 (‘Affected protein function'); a score nearer to 0 indicates more severe damage. PolyPhen2 (http://genetics.bwh.harvard.edu/pph2/) assigned a score of 0.953 (‘Possibly damaging'); a score nearer to 1 indicates more severe damage.

### Database search for proteins that interact with β-catenin

To date, in the BioGRID database^48^, 237 and 69 β-catenin interacting proteins have been archived in humans (http://thebiogrid.org/107880/summary/homo-sapiens/ctnnb1.html) and mouse studies (http://thebiogrid.org/198512/summary/mus-musculus/ctnnb1.html), respectively. The human and mouse β-catenin protein share 100% amino acid sequence identity. However, we should carefully consider proteins for which the interaction domains are uncharacterized or those also contain differences in the armadillo repeats.

## Author Contributions

T.M. and Y.G. wrote the main text. T.M. conducted most of the experiments in this study. Y.I. genotyped the *β-catenin^C429S^* strain and all *β-catenin* gene exons in the *β-catenin^C429S^* strain genome (Table S1). K.K. discovered the point mutations in the *β-catenin* gene (Tables S1, S2). HiKa, HaKo and S.W. conducted *in vitro* fertilization and embryonic transfers (Table S3). H.T. and T.N. assisted with the histological analysis (Figs. 1–4). Y.N. totalled the numbers of genotypes recorded in our mouse database (Tables S4, S5). S.M. and R.F. made public database search to show how significant the C429S mutation. All authors reviewed the manuscript.

## Supplementary Material

Supplementary Information Supplementary information
