# Supplementary material for: β-cateninC429S mice exhibit sterility consequent to spatiotemporally sustained Wnt signalling in the internal genitalia
Source: Sci Rep. 2014 Nov 7;4:6959. doi: 10.1038/srep06959 (PMC4223658; doi:10.1038/srep06959)
Supplement: Supplementary Information [file srep06959-s1.pdf]

Supplementary information

β-catenin^{C429S} mice exhibit sterility consequent to spatiotemporally sustained Wnt signalling in the internal genitalia

Takuya Murata[1]*, Yuichi Ishitsuka[1], Kumiko Karouji[2], Hideki Kaneda[3], Hideaki Toki[4], Yuji Nakai[1], Shigeru Makino[1], Ryutaro Fukumura[1], Hayato Kotaki[1], Shigeharu Wakana[3], Tetsuo Noda[4], and Yoichi Gondo[1]

[1]Mutagenesis and Genomics Team, RIKEN BioResource Center, Tsukuba, Ibaraki, Japan. [2]Population and Quantitative Genomics Team, RIKEN Genomic Sciences Center, Yokohama, Kanagawa, Japan. [3]Japan Mouse Clinic, RIKEN BioResource Center, Tsukuba, Ibaraki, Japan. [4]Team for Advanced Development and Evaluation of Human Disease Models, RIKEN BioResource Center, Tsukuba, Ibaraki, Japan.

The supplementary data contain Figures S1–S5 and Tables S1–S5 as well as the supplementary methods and references.

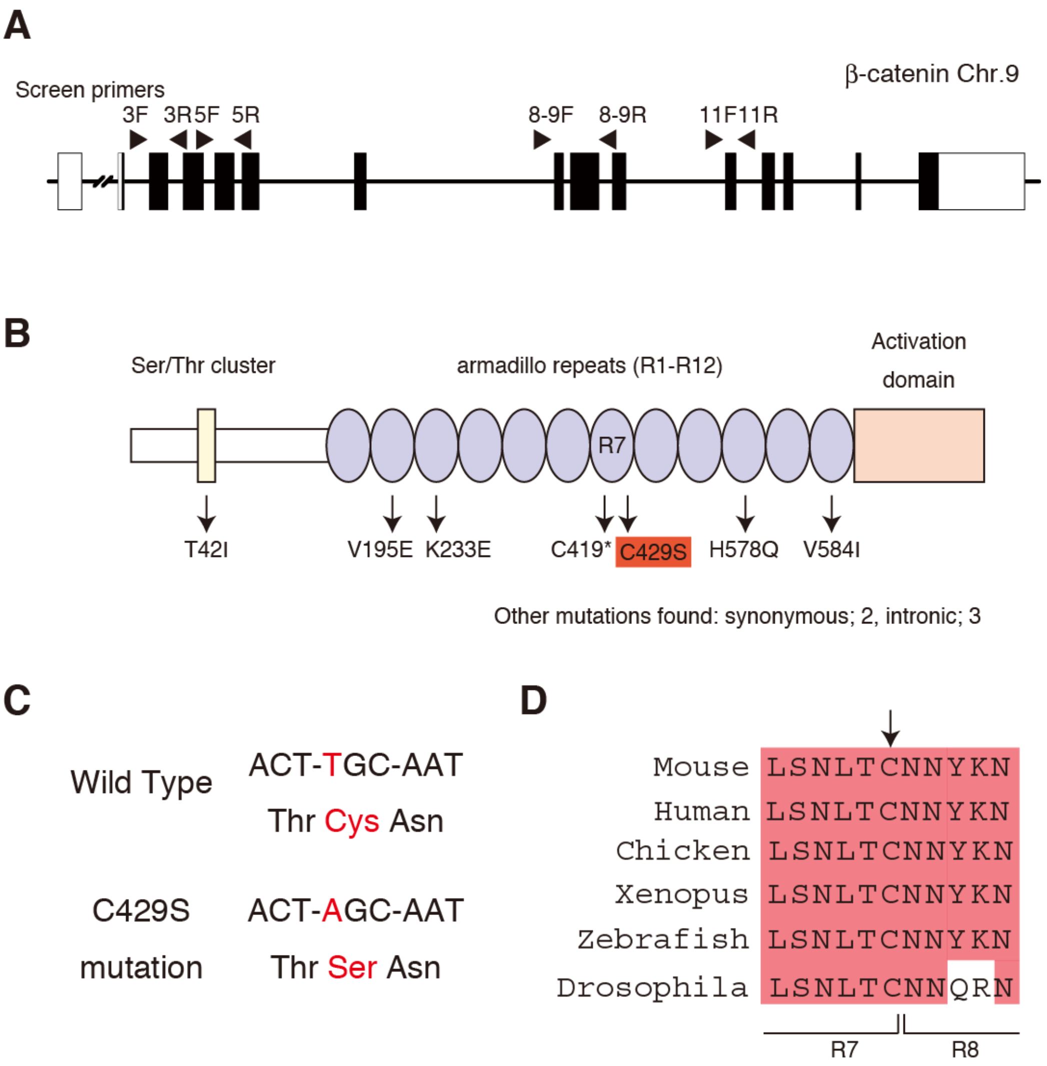

Figure S1: The *β-catenin*^{C429S} mutation was discovered by a high-throughput mutation screening[5] of an ENU-mutagenized male genomic DNA library (A) Schematic representation of the mouse *β-catenin* gene. Arrowheads indicate the primer pairs used for the high-throughput screening (Table S1). (B) Schematic illustration of the 12 mutations. The C429S missense mutation is located in the seventh armadillo repeat. N-terminal Ser/Thr clusters are essential for the regulation of Wnt/β-catenin signalling. Two synonymous and three intronic mutations were also found. (C) The T to A transverse mutation substitutes the cysteine 429 residue to serine. (D) Alignment of the Cys429 and flanking five amino acid sequences. Cys429 is well conserved from humans to *Drosophila*.

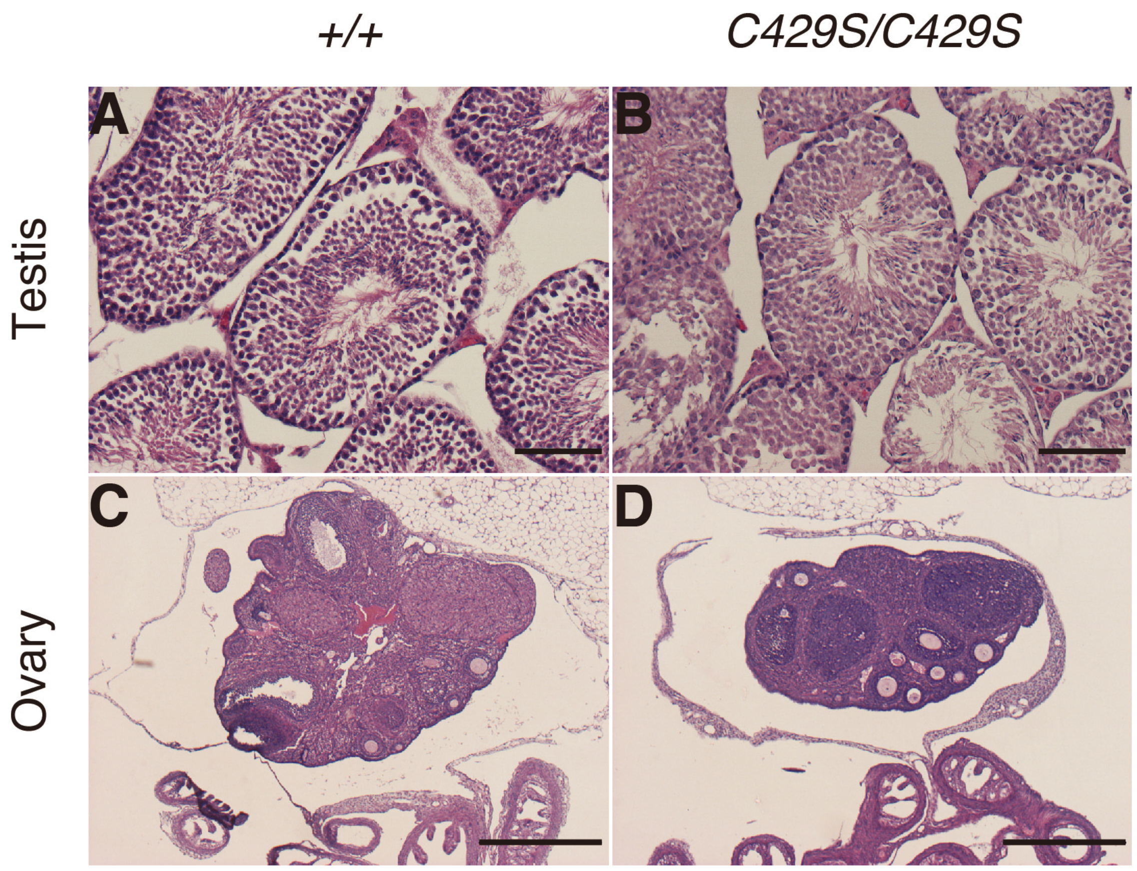

Figure S2: No obvious abnormalities were found in the gonads of *β-catenin*$^{C429S/C429S}$ mice Histological sections of the testis (A, B) and ovary (C, D). No obvious abnormalities were found in the homozygotes. Scale bars: 0.1 mm (A, B) and 0.5 mm (C, D).

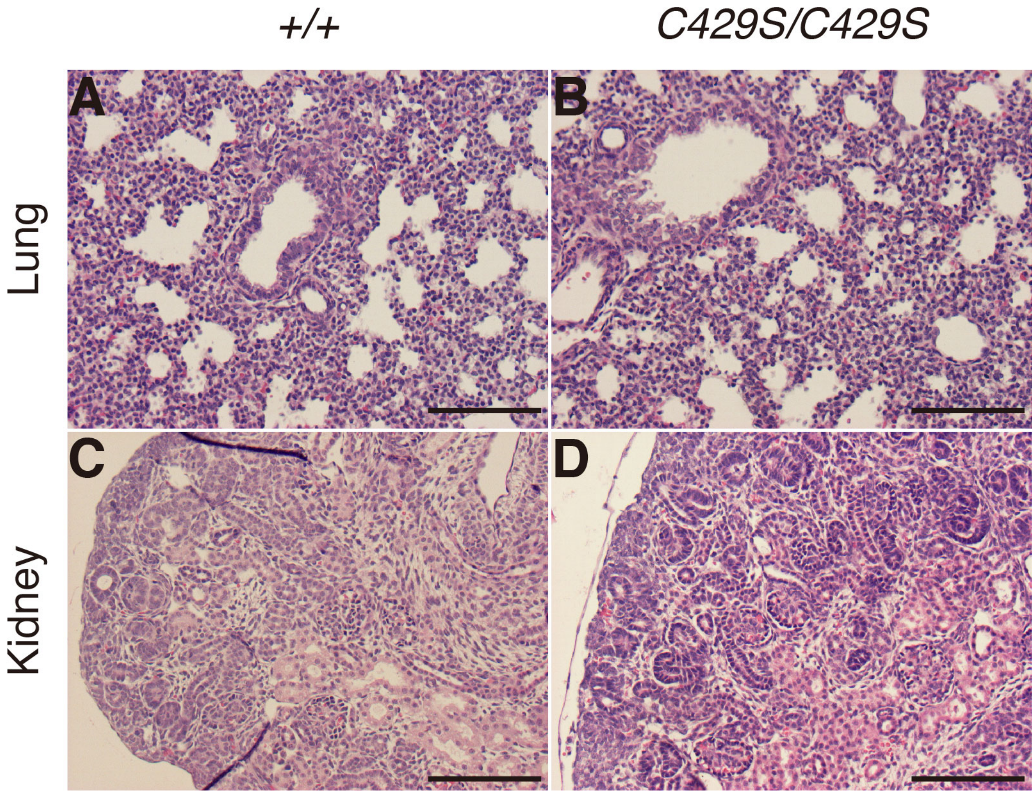

Figure S3: No obvious abnormalities were found in ductal organs other than the seminal vesicle Histological sections of the lung (A, B) and kidney (C, D). Wnt/β-catenin is involved in the regulation of ductal branching and patterning in these organs[6,7,8,9]. Scale bars: 0.1 mm.

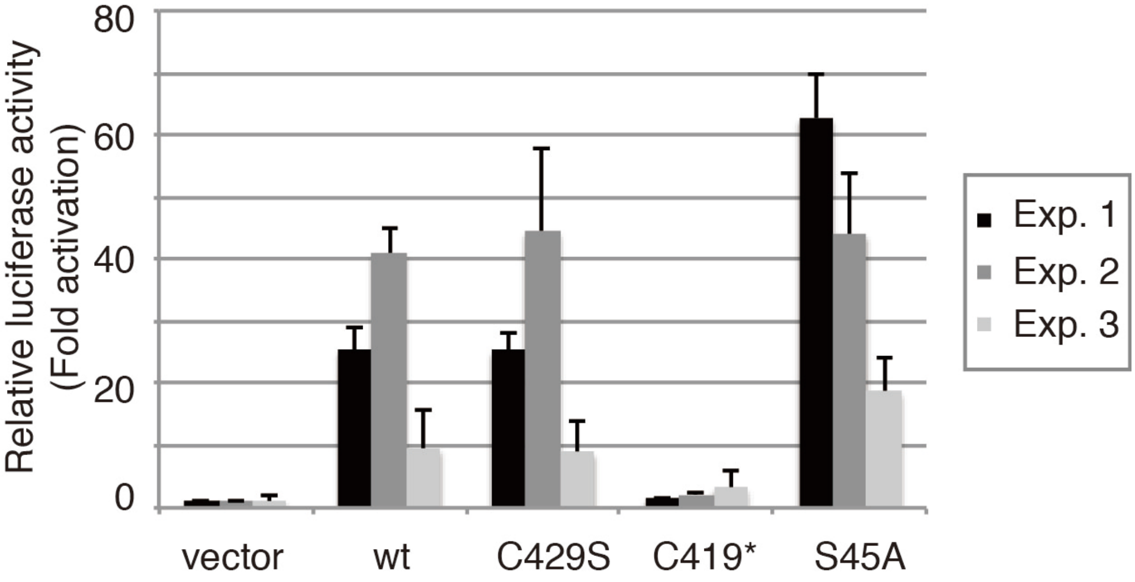

Figure S4: The transcriptional activities of β-catenin^{C429S} and the wild-type protein in a cultured cell line were nearly the same A luciferase reporter assay involving a Wnt/β-catenin signal reporter, TOPFLASH[3] and *β-catenin* expression plasmids. In this experiment, we implemented not only wild-type *β-catenin* but also the C429S, C419* (negative control that was found in Fig. S1) and S42A (constitutive active control) mutants. β-catenin escapes from default degradation upon Wnt ligand stimulation[10] and the S42A mutation prevents this process[1]. The data are shown in terms of 'fold activation', defined as the relative activity versus that of the mock (vector only) control. The same experiments were repeated three times. The results are illustrated by distinct dark bars (Exp. 1–3).

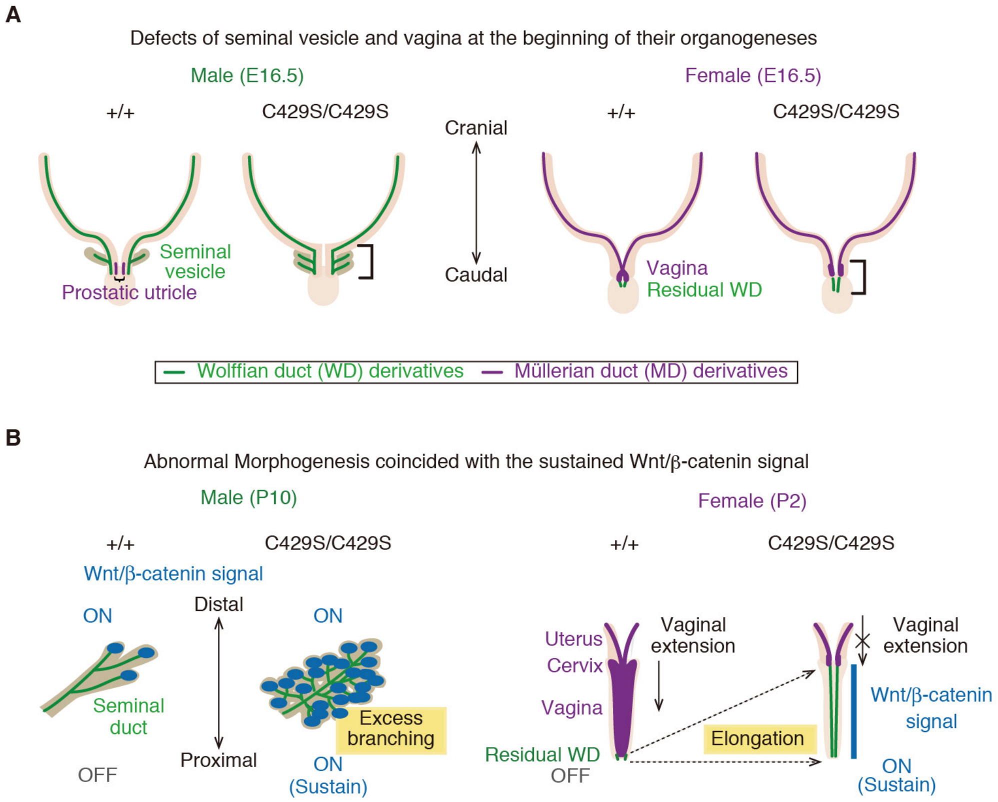

Figure S5: Abnormally sustained Wnt/β-catenin signalling may affect Wolffian duct morphogenesis, directly and indirectly leading to anomalies of the seminal vesicle in males and vagina in females, respectively (A) Abnormal morphologies were exhibited from the beginning of organogenesis of both the seminal vesicle and vagina (E16.5). In males, ins-TOPGAL revealed (Fig. 3E) the bent Wolffian duct (white arrow) and extra seminal duct bud (black bracket) as well as the disappearance of the prostatic utricle (black arrow). In females, ins-TOPGAL revealed an unfused Müllerian duct (white arrowhead in Fig. 4C) and elongated Wolffian duct (white arrows in Fig. 4C, E). (B) This sustained Wnt/β-catenin signalling coincided with the abnormal morphogenesis. At P10, the sustained ins-TOPGAL expression was uniformly observed in the excessively branched seminal ducts (Fig. 3K). At P2, the sustained Wnt/β-catenin signalling was observed in the elongated ‘residual’ Wolffian duct in females (Fig. 4E). The Wolffian duct appears to have physically prevented vaginal extension. Otherwise, given that the residual Wolffian duct plays a role of a ‘helper’in vaginal extension[11], the duct may hamper vaginal extension via tissue interactions. Both possibilities suggest an indirect effect of the abnormal Wolffian duct.

Table S1 | Primers used for the high-throughput screening of ENU mutations in the *β-catenin* gene and sequencing of all coding exons with flanking introns

Name	Sequence	Name	Sequence
Ex 2F	TGTATTTTTAGTGTATGCCATGGTG	Ex 2R	ATTATTTCACCAAGCCAGTGGTAGT
Ex 3F*	CTCTCCTTGGCTGCCTTTCTAACAG	Ex 3R*	CAACACTCACTAGGCATGTGGGACTC
Ex 4F	CATCACTGCTTACCTGGTCCTCATCG	Ex 4R	ACTATCAGGATGACGCGGAACTTGC
Ex 5F*	ACTATCAGGATGACGCGGAACTTGC	Ex 5R*	GGCGTAGAACAGTACAGAATCCACTGG
Ex 6F	CTTTAAGTCTGGTGGCATCCCAGC	Ex 6R	ATCTTTGAGCCTGTGTGTGTGTGCG
Ex 7F	GTGGATCAGAAGGGTCCAGAGAGATAC	Ex 7R	CTAGATTCACAGGGCTGCTAGTGAGG
Ex 8-9F*	ATGTCTGACTTTGCAGGTGGGATGC	Ex 8-9R*	GTGTTGAGTGAAATGGACGCTTCGG
Ex 10F	CATCCCACTGGCCTCTGATAAAGG	Ex 10R	GGTAGAGACAGCACATGGAGATTGC
Ex 11F*	TATACTCAGCGGAAGGGACCCTCC	Ex 11R*	CGCCCTTCTCCTCAGGCTATAGTACC
Ex 12-13F	TAGGTCTTGTTTCTGTGTTTTCTCC	Ex 12-13R	CTCATGCTAGCTGGAGTTAAGAGC
Ex 14F	TTTTATGTCCTTCTTGTACCCATTT	Ex 14R	AGTCTGATCTGCCTGTCACAGAT
Ex 15F	TGGCAAGATGTCCTTACCACAGTTA	Ex 15R	TGTTTGGTGGCAGATTTACAAACAG

Primers used for high-throughput screening are indicated with asterisks. All primers were used to sequence all of the coding exons with flanking introns. All sequence data will be disclosed upon request.

Table S2 | List of *β-catenin* (also known as *Ctnnb1*) point mutation-bearing mouse lines deposited in the RIKEN BioResource Center

Mutant allele	Mutation type	Detail	RIKEN BRC Deposit Number	Chromosomal location (GRCm38/mm10)	Mutation
$\textit{Ctnnb1}^{Rgsc01880}$	Missense	C429S	RBRC-GD000125	Chr9:120955428	T to A
$\textit{Ctnnb1}^{Rgsc01834}$	Missense	T42I	RBRC-GD000129	Chr9:120950633	C to T
$\textit{Ctnnb1}^{Rgsc01187}$	Intronic		RBRC-GD000130	Chr9:120950804	C to A
$\textit{Ctnnb1}^{Rgsc01837}$	Missense	V195E	RBRC-GD000131	Chr9:120951355	T to A
$\textit{Ctnnb1}^{Rgsc00962}$	Synonymous	C573C	RBRC-GD000132	Chr9:120957142	T to C
$\textit{Ctnnb1}^{Rgsc00879}$	Nonsense	C419*	RBRC-GD000133	Chr9:120955400	T to A
$\textit{Ctnnb1}^{Rgsc01881}$	Synonymous	N483N	RBRC-GD000134	Chr9:120955592	T to C
$\textit{Ctnnb1}^{Rgsc01937}$	Missense	H578Q	RBRC-GD000135	Chr9:120957157	C to A
$\textit{Ctnnb1}^{Rgsc01021}$	Intronic		RBRC-GD000136	Chr9:120957038	A to G
$\textit{Ctnnb1}^{Rgsc01938}$	Missense	V584I	RBRC-GD000137	Chr9:120957173	G to A
$\textit{Ctnnb1}^{Rgsc01940}$	Missense	K233E	RBRC-GD000138	Chr9:120951468	A to G
$\textit{Ctnnb1}^{Rgsc01943}$	Intronic		RBRC-GD000139	Chr9:120951226	A to T

List of mouse lines discovered by high-throughput screening of the RIKEN ENU Mutant Mouse Library. All mouse lines are available from the RIKEN BioResource Center.

Table S3 | The mutant sperm and oocytes were functionally normal

Male genotype	Female genotype	Fertility	Birth rate
C429S/C429S #1	C57BL/6J (n = 4.5)	91% (165/181)	20% (19/96)
C429S/C429S #2	C57BL/6J (n = 4.5)	91% (116/127)	40% (20/48)
C429S/C429S #3	C57BL/6J (n = 4)	95% (121/127)	20% (20/96)
C429S/C429S #1–3	C57BL/6J (n = 13)	92% (402/435)	25% (59/240)
+/+ #1	C57BL/6J (n = 4.5)	95% (199/210)	24% (17/72)
+/+ #2	C57BL/6J (n = 4)	94% (133/142)	42% (20/48)
+/+ #3	C57BL/6J (n = 4)	92% (499/540)	28% (61/216)
+/+ #1–3	C57BL/6J (n = 12.5)	92% (499/540)	28% (61/216)
C57BL/6J (n=1)	C429S/C429S #1	96% (45/47)	22% (15/69)
	C429S/C429S #2	100% (24/24)	
	C429S/C429S #1–2	97% (69/71)	22% (15/69)
	+/+ #1	79% (38/48)	22% (28/126)
	+/+ #2	89% (50/56)	
	+/+ #3	94% (32/34)	
	+/+ #4	100% (6/6)	
	+/+ #1–4	89% (126/164)	22% (28/126)

The sperm and oocytes from wild-type and homozygous mice were used for *in vitro* fertilization (IVF) and subsequent embryonic transfer experiments. Oocytes were collected via superovulation from mice as young as 4 weeks of age. Four or 4.5 C57BL/6J females were used per each homozygous male. The collected oocytes were fertilized with sperm from three homozygous and three wild-type males and then subjected to embryonic transfer. A value of 0.5 indicates that the oocytes were collected from a single ovary. Collected sperm were carefully observed according to the sperm count, motility and morphology; no significant differences were observed between the wild-type and homozygous samples.

One C57BL6/J male was used for IVF with oocytes from two homozygous and four wild-type females. In the subsequent embryo transfer, two-celled embryos from homozygous and wild-type females were pooled into one sample each.

‘Fertility’ indicates the number of two-cell-stage embryos from the total oocytes used for IVF. ‘Birth rate’ indicates the number of pups from transferred two-cell-stage embryos.

Table S4 | Genital malformations were recessive in males and semi-dominant in females

	Male			Female		
	+/+	*C429S*/+	*C429S*/*C429S*	+/+	*C429S*/+	*C429S*/*C429S*
Number of genotypes	5	8	2	4	10	2
Number of phenotypes	0	0	2	0	2	2

Twentieth-generation *β-catenin*$^{C429S/+}$ males and females were used for intercrossing. Genital malformations, duplications of extra seminal vesicle in males and vaginal atresia in females, were counted as genotypes in the weaned pups.

Table S5 | Segregation of the *β-catenin*^{C429S} genotypes by intercrossing

Male			Female		
+/+	C429S/+	C429S/C429S	+/+	C429S/+	C429S/C429S
123	215	69	100	166	50

The total number of genotypes per gender obtained by intercrossing *β-catenin*^{C429S} mice in the fifth to twenty-first generations. The chi-square test indicated significantly low viability of the *β-catenin*^{C429SC429S} genotype ($p < 0.05$).

Supplementary methods

Plasmids

Human Ultimate™ ORF LITE Clone *β-catenin* cDNA was purchased from Invitrogen (Carlsbad, CA, USA). The human and mouse β-catenin proteins share 100% amino acid similarity. The QuickChange Site-Directed Mutagenesis Kit (Agilent Technologies, Santa Clara, CA, USA) was used to introduce the C429S, C419* [nonsense mutation found at the same time as C429S (Fig. S1); negative control] and S42A (constitutive active mutation; positive control[1]) point mutations. The following primers were used: C429S-F, 5'-GGAATTCTTTCTAACCTCACTAGCAATAATTATAAGAAC-3' and C429S-R, 5'-GTTCTTATAATTATTGCTAGTGAGGTTAGAAAGAATTCC-3'; C419*-F, 5'-GATATAAATGTGGTCACCTGATGAGCTGGAATTCTTTCTAACC-3' and C419*-R, 5'-GGTTAGAAAGAATTCCAGCTCATCAGGTGACCACATTTATATC-3'; S45A-F, 5'-CCACTACCACAGCTCCTGCTCTGAGTGGTAAAGG-3' and S45A-R, 5'-CCTTTACCACTCAGAGCAGGAGCTGTGGTAGTGG-3'. The C429S, C419*, S42A and wild-type cDNA were cloned into pcDNA™ 3.1/nV5-DEST Mammalian Expression Vector via Gateway® technology (Invitrogen, Carlsbad, CA, USA).

Luciferase assay

HilyMax (Wako, Japan) was used for lipofection, according to the manufacturer's instructions. The day before transfection, 1.6×10^5 cells/well were seeded in a 24-well plate. The amounts of plasmid added per well were as follows: pcDNA-*β-catenin* (wt or mt), 100 ng; pCMX-βGal[2] for transfection normalization, 10 ng; TOPFLASH[3], 10 ng and pCMX[2] for balance, 130 ng. At 24 h after transfection, the cells were collected and subjected to luciferase and β-galactosidase assays as described previously[4]. Simultaneous transfections were performed in triplicate. The relative luciferase activity (luciferase/β-galactosidase) was calculated as the average of the triplicate transfections along with the standard deviation.

Supplementary reference

1. Wang S, Jones KA. CK2 controls the recruitment of Wnt regulators to target genes in vivo. *Curr Biol* **16**, 2239-2244 (2006).

2. Umesono K, Murakami KK, Thompson CC, Evans RM. Direct repeats as selective response elements for the thyroid hormone, retinoic acid, and vitamin D3 receptors. *Cell* **65**, 1255-1266 (1991).

3. Roose J*, et al.* The Xenopus Wnt effector XTcf-3 interacts with Groucho-related transcriptional repressors. *Nature* **395**, 608-612 (1998).

4. Murata T, Nitta M, Yasuda K. Transcription factor CP2 is essential for lens-specific expression of the chicken alphaA-crystallin gene. *Genes Cells* **3**, 443-457 (1998).

5. Sakuraba Y*, et al.* Molecular characterization of ENU mouse mutagenesis and archives. *Biochem Biophys Res Commun* **336**, 609-616 (2005).

6. Mucenski ML*, et al.* beta-Catenin is required for specification of proximal/distal cell fate during lung morphogenesis. *J Biol Chem* **278**, 40231-40238 (2003).

7. Bridgewater D*, et al.* Canonical WNT/beta-catenin signaling is required for ureteric branching. *Dev Biol* **317**, 83-94 (2008).

8. Marose TD, Merkel CE, McMahon AP, Carroll TJ. Beta-catenin is necessary to keep cells of ureteric bud/Wolffian duct epithelium in a precursor state. *Dev Biol* **314**, 112-126 (2008).

9. Miller RK, McCrea PD. Wnt to build a tube: contributions of Wnt

signaling to epithelial tubulogenesis. *Dev Dyn* **239**, 77-93 (2010).

10. Valenta T, Hausmann G, Basler K. The many faces and functions of beta-catenin. *EMBO J* **31**, 2714-2736 (2012).

11. Drews U. Helper function of the Wolffian ducts and role of androgens in the development of the vagina. *Sex Dev* **1**, 100-110 (2007).
